# How to eat, drink and speak on non-invasive ventilation

**DOI:** 10.1177/14799731211061156

**Published:** 2021-12-21

**Authors:** William Kinnear, Karah Dring, Katherine Kinnear, Jane Hansel, Milind Sovani

**Affiliations:** 1 Department of Sport Science, School of Science and Technology, 6122 Nottingham Trent University, Nottingham, UK; 2 12212 University of Warwick, Coventry, UK; 3 Queens Medical Centre Campus, 9820 Nottingham University Hospitals, Nottingham, UK

**Keywords:** Swallowing, speech, non-invasive ventilation, neuromuscular disorders

## Abstract

We report our observations on six individuals with non-bulbar neuromuscular disorders using non-invasive ventilation (NIV), who were able to maintain adequate hydration and nutrition orally despite being ventilator-dependant. All had severe respiratory muscle weakness, with a vital capacity less than 500 mL and cough peak flow rate less than 250 L/min. Their median (range) age was 49 (23–64) years; they had been on NIV for 8 (2–24) years. We compared them with an age- and sex-matched normal control. Individuals with neuromuscular disorders needed to chew each mouthful of food significantly more times (median 44, range 18–120 chews) than normal controls (median 15, range 10–20 chews). They took longer to completely swallow a mouthful of food (median 37, range 24–100 s) compared to normal controls (median 14.5, range 10–21 s). Multiple swallows for each mouthful were seen in all neuromuscular individuals, but in only one normal control. Two individuals coughed after swallowing; both these subjects were clinically stable at the time of the study. The median number of NIV breaths associated with chest expansion for each mouthful was 11 (range 5–49). All subjects blocked some NIV breaths whilst eating. Before swallowing, they always waited until the expiratory phase of the NIV breath was complete; no post-swallow expiration was seen, whereas normal subjects invariably exhibited post-swallow expiration. All individuals were able to block several ventilator breaths whilst swallowing un-thickened liquids. The median (range) number of words between breaths was 5 (4–7) for the neuromuscular individuals on NIV, significantly fewer than 11 (8–13) for the matched controls. Eating, drinking and speaking are possible whilst on NIV. Use of cough-assist after eating is recommended, given the likelihood of silent aspiration.

## Introduction

The ability to eat, drink and speak whilst using non-invasive ventilation (NIV) can have a positive effect on quality of life. We have studied individuals with non-bulbar neuromuscular disorders, who were able to maintain adequate hydration and nutrition orally despite being ventilator-dependant. They had not received any specific training on how to eat, drink and speak during NIV.

## Methods

From our database we identified six NIV-dependant individuals with non-bulbar neuromuscular disorders (Table 1). All had severe respiratory muscle weakness, with a vital capacity less than 500 mL and cough peak flow rate less than 250 L/min. Their median (range) age was 49 (23–64) years; they had been on NIV for 8 (2–24) years. None had a history of oro-pharyngeal dysphagia, nor clinical signs of bulbar weakness.

**Table 1.** Details of six individuals with non-bulbar neuromuscular disorders who were able to eat and drink whilst using NIV.

	Gender	Age in years	Diagnosis	Ambulatory status	Years on NIV	Food	Drink
1	Male	55	Limb girdle muscular dystrophy	Ambulant with frame	8	Sandwich, with crust	Water
2	Male	29	Duchenne muscular dystrophy	Non-ambulant	9	Sandwich, without crust	Orange squash
3	Male	64	Amyotrophic lateral sclerosis	Non-ambulant	2	Sandwich, with crust	Water
4	Female	43	Osteogenesis imperfecta with cervical myelopathy	Non-ambulant	14	Minced dish with mashed potatoes	Tea
5	Male	23	Congenital myopathy	Non-ambulant	8	“Soft” casserole, with mashed potatoes	Orange squash
6	Female	61	Spinal muscular atrophy	Ambulant with frame	24	Minced dish, with mashed potatoes	Tea

Note: NIV: non-invasive ventilation.

We recorded videos in their own homes as they ate their normal lunchtime meal, whilst continuing on NIV using a nasal mask or nasal pillows. The video camera was positioned to record eating and drinking from the side, sufficiently close to the subject that the phase of the ventilator and airflow through the expiratory valve (or port) could be heard on the recording. The recordings were analysed independently by two of the authors. Although repeatability was not formally assessed prior to the study, agreement between the observers was good and the mean of their scores for chews, times and swallows were used in subsequent analysis. The presence of a swallow was detected by laryngeal elevation and cessation of mandibular chewing motion. We observed the total time taken to swallow a mouthful of food, together with the number of chews and swallows for each mouthful. Completion of swallowing for each mouthful was confirmed by visual inspection of the oral cavity. Each individual was also observed drinking fluids, without the addition of thickeners. Whilst speaking, we counted the number of words spoken between breaths delivered by the ventilator.

For each individual with a neuromuscular disorder, we also made recordings in their own home of one age- and sex-matched matched normal control eating food of similar consistency, swallowing un-thickened fluids and speaking. All normal controls had spirometry values within the normal range and were non-smokers. (It should be noted that all of our observations could be subject to observer bias, given that the assessors could always see whether or not the individual was using NIV.)

All individuals were on pressure-control rather than triggered modes of NIV. Two were using bi-level (inspiratory and expiratory) positive airway pressure; the remainder were on positive airway pressure during inspiration only, but with an expiratory valve. If no chest expansion was seen in time with the positive pressure delivered by the ventilator, we designated that breath to have been “blocked” by voluntary closure of the upper airway. We noted the timing of swallowing with regard to breathing, looking for post-swallow expiration.

Comparisons for number of chews and total time to swallow each mouthful of food between the two groups were made using Man Whitney U tests, taking 0.05 as the level of statistical significance. Verbal consent was obtained from each subject, in the presence of an independent witness. The study was approved by the clinical ethics committee of Nottingham University Hospitals.

## Results

**Figure 1.**
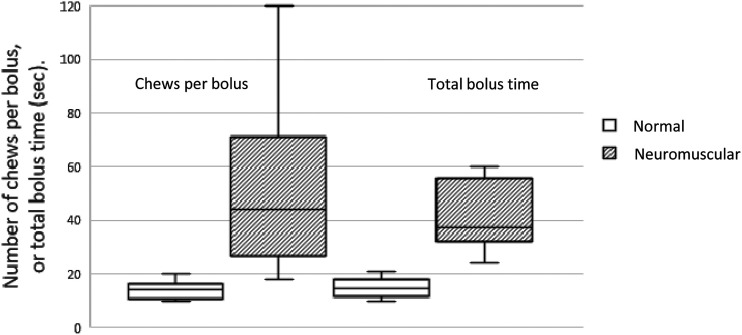
Number of chews and total time to swallow each bolus in subjects with neuromuscular disorders whilst on non-invasive ventilation, compared to normal subjects.

The individuals with neuromuscular disorders needed to chew each mouthful of food significantly more times (median 44, range 18–120 chews) than normal controls (median 15, range 10–20 chews). They took longer to completely swallow a mouthful of food (median 37, range 24–100 s) compared to normal controls (median 14.5, range 10–21 s) (Figure 1).

Multiple swallows for each mouthful were seen in all of the individuals with neuromuscular disorders, with a median of two swallows per mouthful, but in only one control. Two individuals, but no controls, coughed after swallowing; both were clinically stable at the time of the study, with no clinical evidence of aspiration pneumonia.

The median number of NIV breaths associated with chest expansion for each bolus was 11 (range 5–49). All subjects blocked some NIV breaths whilst swallowing solids. Before swallowing, they always waited until the expiratory phase of the NIV breath was complete; no post-swallow expiration was seen, whereas normal subjects invariably exhibited post-swallow expiration. All individuals were able to block several ventilator breaths whilst swallowing liquids. (None of the individuals used thickeners in their drinks.)

The median (range) number of words between breaths was 5 (4–7) for the neuromuscular subjects on NIV, significantly fewer than 11 (8–13) for the matched control subjects.

## Discussion

Individuals with severe respiratory muscle weakness who were able to breath spontaneously can be managed safely at home.^1–3^ Many will require an enteral feeding tube, but if they are able to manage without NIV for a short time, a mouthpiece can be used to deliver a few NIV breaths at intervals during a meal.^4^ Some find it easier to continue with NIV whilst they eat and speak,^5,6^ learning largely by trial and error how best to manage this combination. On the basis of the observations made in this study, we have drawn up suggestions as to how these skills might be taught, we hope will be of value to those encountering this problem for the first time (Tables 2 and 3).

**Table 2.** Protocol for teaching how to eat whilst on NIV.

1. Explanation of dual function of pharynx (to conduct air into the trachea and food into the oesophagus)
2. Without eating, learning to block ventilator breaths by upper airway closure
3. Discussion of choice of food consistency
4. Using a very small food bolus, extend duration of chewing over several breaths
5. Swallow this bolus between NIV breaths
6. Block following breath
7. Subdivide a larger bolus into several swallows
8. Practice use of cough assist after meal

Note: NIV: non-invasive ventilation.

**Table 3.** Protocol for teaching how to speak whilst on non-invasive ventilation.

1. Explanation of phonation by expiration through vocal cords
2. Repeated phonation with single syllable throughout respiratory cycle, to demonstrate loss of volume during inspiration
3. Repeated phonation with single syllable in expiration only, counting number of syllables with each breath
4. Practice use of short phrases with this number of syllables
5. Trial of breath stacking, with laryngeal closure at end of inspiration until beginning of next ventilator breath, to see if this increases voice volume

Our individuals with neuromuscular disorders were slow eaters, taking their time to chew their food many times. This is likely to have been influenced by their choice of food, the strength of their oropharyngeal muscles and the necessity to co-ordinate with NIV. Piecemeal swallowing was invariable in our neuromuscular subjects,^7^ block the ventilator breath whilst swallowing. Without video-fluoroscopy, we cannot ascertain exactly how they kept food forward in their mouth whilst NIV continued uninterrupted, nor the mechanism of blocking NIV breaths.

Our study population found it easy to work around the predictable pattern of a pressure-control mode of NIV, which delivers breaths at a fixed rate. Although patient-triggered pressure support can be more comfortable, back-up breaths are less easy to anticipate, and non-inspiratory triggering of the ventilator during chewing and swallowing can be problematic. External switches can be used to pause the ventilator for swallowing.^7–11^ Positive pressure in the lower airway can interfere with normal swallowing,^12^ but both of our subjects on bi-level modes of NIV were able to swallow effectively.

In normal individuals, swallowing is preceded by inspiration and followed by expiration, this sequence presumably ensuring that any food residue in the upper airway is cleared before the next inspiration.^13,14^ Post-swallow expiration on NIV has been shown in both normal subjects and chronic obstructive pulmonary disease.^10,11^ The absence of post-swallow expiration on NIV has been reported previously in neuromuscular disorders.^7^ It would be of interest to see if individuals with neuromuscular disorders could learn to swallow immediately after the inspiratory phase of NIV, holding air in their lungs which could then be exhaled after the swallow in order to clear the upper airway.

Two of our individuals with neuromuscular disorders coughed during their meal, and we cannot exclude “silent” aspiration in the others. The use of cough-assist after a meal would be prudent in this situation.^5^

Mouthpiece ventilation, by providing an initial large inspiration, increases speech volume.^15^ Use of a bi-level NIV with a nasal mask can lead to muffled speech with a nasal quality. With pressure-control NIV, sentences will be interrupted by ventilator breaths, but our subjects found it easy to communicate in short phrases. This approach was easily learnt by our subjects.

In conclusion, individuals with neuromuscular disorders are able to eat, drink and speak whilst using NIV. We make suggestions as to how these skills can be taught. Use of cough-assist is recommended, given the likelihood of silent aspiration. Future studies should look at the timing of swallowing, to see if post-swallow expiration can be achieved in individuals with neuromuscular disorders. Further investigation is also needed to look at swallowing during NIV in individuals with other disorders such as COPD, and those more recently started on NIV.
